# Supplementary material for: The Effectiveness and Safety of Acupuncture on Suicidal Behavior: A Systematic Review
Source: Healthcare (Basel). 2023 Mar 27;11(7):955. doi: 10.3390/healthcare11070955 (PMC10094566; doi:10.3390/healthcare11070955)
Supplement: Supplementary file 1 [file healthcare-11-00955-s001.zip › Supplement Table S2.pdf]

Supplement Table S2. Search strategies and the results (search date: 2022.09.07.)

Medline via PubMed

	Searches	Results
#1	Suicide[MH] OR “Self Mutilation”[MH] OR self-harm OR self-poisoning OR self-injur* OR “Self Mutilation” OR “attempted suicide” OR suicid*	127,217
#2	Acupuncture[MH] OR Acupressure[MH] OR “Acupuncture Therapy”[MH] OR “Acupuncture Points”[MH] OR acupunct* OR electroacupunct* OR electro-acupunct* OR acupoint* OR acupressure OR auriculotherapy	41,266
#3	#1 AND #2	**44**

EMBASE via Elsevier

	Searches	Results
#1	‘suicidal behavior’/exp OR ‘suicide’/exp OR ‘automutilation’/exp OR self-harm OR self-poisoning OR self-injur* OR “Self Mutilation” OR “attempted suicide” OR suicid*	174,074
#2	‘acupuncture’/exp OR ‘acupuncture point’/exp OR ‘electroacupuncture’/exp OR ‘acupunct*’ OR ‘electroacupunct*’ OR ‘electro-acupunct*’ OR ‘acupoint*’ OR ‘acupressure’ OR ‘auriculotherapy’	64,752
#3	#1 AND #2	**180**

CENTRAL

	Searches	Results
#1	MH descriptor: [Suicide] explode all trees	1,503
#2	MH descriptor: [Self Mutilation] explode all trees	37
#3	self-harm OR self-poisoning OR self-injur* OR “Self Mutilation” OR “attempted suicide” OR suicid*	8,740
#4	MH descriptor: [Acupuncture] explode all trees	164
#5	MH descriptor: [Acupressure] explode all trees	428
#6	MH descriptor: [Acupuncture Therapy] explode all trees	5,344
#7	MH descriptor: [Acupuncture Points] explode all trees	2,272
#8	acupunct* OR electroacupunct* or electro-acupunct* OR acupoint* OR acupressure OR auriculotherapy	22,515
#9	(#1 OR #2 OR #3) AND (#4 OR #5 OR #6 OR #7 OR #8) in Trials	**27**

CINAHL via EBSCO

	Searches	Results
#1	SU Suicide OR SU Self Mutilation OR TX self-harm OR TX self-poisoning OR TX self-injur* OR TX Self Mutilation OR TX attempted suicide OR TX suicid*	92,437
#2	SU Acupuncture OR SU Acupressure OR SU Acupuncture Therapy OR SU Acupuncture Points OR TX acupunct* OR TX electroacupunct* OR TX electro-acupunct* OR TX acupoint* OR TX acupressure OR TX auriculotherapy	38,370
#3	#1 AND #2	**690**

AMED via EBSCO

	Searches	Results
#1	SU Suicide OR SU Self Mutilation OR TX self-harm OR TX self-poisoning OR TX self-injur* OR TX Self Mutilation OR TX attempted suicide OR TX suicid*	1,987
#2	SU Acupuncture OR SU Acupressure OR SU Acupuncture Therapy OR SU Acupuncture Points OR TX acupunct* OR TX electroacupunct* OR TX electro-acupunct* OR TX acupoint* OR TX acupressure OR TX auriculotherapy	12,505
#3	#1 AND #2	**6**

PsycARTICLES via ProQuest

	Searches	Results
#1	SU(Suicide) OR SU("Self Mutilation") OR self-harm OR self-poisoning OR self-injur* OR "Self Mutilation" OR "attempted suicide" OR suicid*	20,175
#2	SU(Acupuncture) OR SU(Acupressure) OR SU("Acupuncture Therapy") OR SU("Acupuncture Points") OR acupunct* OR electroacupunct* OR electro-acupunct* OR acupoint* OR acupressure OR auriculotherapy	408
#3	#1 AND #2	**120**

CNKI

	Searches	Results
#1	(SU='自杀'+'自伤'+'suicide'+'self-harm') AND (SU='针'+'鍼'+'穴位按压'+'耳穴'+'acupuncture'+'acupressure'+'auriculotherapy')	**13**

WanFang data

	Searches	Results
#1	主题:("自杀" or "自伤" or "suicide" or "self-harm") and 主题:("针" or "鍼" or "穴位按压" or "耳穴" or "acupuncture" or "acupressure" or "auriculotherapy") not 主题:(针对)	**515**

VIP

	Searches	Results
#1	M=(自杀 or 自伤 or suicide or self-harm) AND M=(针 or 鍼 or 穴位按压 or 耳穴 or acupuncture or acupressure or auriculotherapy)	**473**

CiNii

	Searches	Results
#1	(自殺 OR 自傷 OR suicide OR self-harm) AND (鍼 OR 圧迫 OR acupuncture OR acupressure OR auriculotherapy)	**151**

OASIS

	Searches	Results
#1	(자살 OR 자해) AND (침 OR 지압)	**0**

KISS

	Searches	Results
#1	(자살 OR 자해) AND (침)	191
#2	(자살 OR 자해) AND (지압)	0
#3	#1 AND #2	**191**

KCI

	Searches	Results
#1	(자살 OR 자해) AND (침)	41

#2	(자살 OR 자해) AND (지압)	0
#3	#1 AND #2	**41**

RISS

	Searches	Results
#1	(자살 OR 자해) AND (침)	63
#2	(자살 OR 자해) AND (지압)	0
#3	#1 AND #2	**63**

KMbase

	Searches	Results
#1	(자살 OR 자해) AND (침 OR 지압)	0
