# Supplementary material for: The Effectiveness and Safety of Acupuncture on Suicidal Behavior: A Systematic Review
Source: Healthcare (Basel). 2023 Mar 27;11(7):955. doi: 10.3390/healthcare11070955 (PMC10094566; doi:10.3390/healthcare11070955)
Supplement: Supplementary file 1 [file healthcare-11-00955-s001.zip › Supplement Table S3.pdf]

Supplement Table S3. Major findings and safety data from included studies.

Study / Country / Study design	Sample size (included→analyzed)	Intervention	Treatment duration (f/u)	Major findings	Safety data
Wang (2014) / China / RCT	61(20:21:20)→55(19:16:20)	TG1: EA+placebo pill; TG2: EA+SSRI; CG1: placebo EA+SSRI	6 wk (4-wk post-treatment)	1. CSRS: The CSRS recordings showed that, over the whole time period of the trial, no patients presented a risk of suicide. (Raw data not reported)	TG1: 2/19 (nausea), 2/19 (fatigue), 1/19 (itchy); TG2: 4/20 (stomach discomfort and nausea), 2/20 (sleepy during the day), 2/20 (menstrual change), 1/20 (forgetfulness); CG: 2/16 (eye discomfort), 4/16 (stomach discomfort and nausea), 1/20 (fatigue)
Yang (2014) and Yang et al. (2020) / China / CCT	60(35:25)→55(32:23)	TG: EA; CG: SSRI	8 wk (4-wk post-treatment)	1. CSRS: The CSRS recordings showed that, over the whole time period of the trial, no patients presented a risk of suicide. (Raw data not reported)	TG: 4/32 (bleeding and hematoma around the site of needling); CG: 13/23 (dizziness, nausea, tremors, palpitations and dry mouth)
Li et al. (2018) / China / RCT	93(31:31:31)→83(29:27:27)	TG1: ROC+AA (magnetotherapy); TG2: ROC+AA (vaccaria seed); CG: ROC for stroke	4 wk (4-wk post-treatment)	1. Item 3 of HAMD-17 (Suicide): 0.52±0.51 to 0.34±0.48 (TG1<CG: $p<0.01$, TG1<TG2: $p<0.01$) / 0.63±0.57 to 1.00±0.39 (TG2<CG: $p<0.01$) / 0.67±0.62 to 1.15±0.66	None
Jin et al. (2019) / China / Before-after study	21→21	Acupuncture	3 mon	1. CER (reduction of HAMD score): 90.48%; 2. Number of patients with suicidal ideation: 21 / 7; 3. BSSI: 38.90±4.43 / 17.38±8.83 ($p<0.05$)	NR
Pirnia et al. (2019) / Iran / single-case experimental study	1	AA+methadone	4 wk	1. Salivary cortisol (unclear unit and the timing of sampling): 30.67±1.23 / 24.50±1.44 ($p<0.05$); 2. Suicidal ideation (unclear assessment tool): Raw data not reported, but described as significantly reduced ($p<0.01$).	NR
Pirnia et al. (2019) / Iran / RCT	24(12:12)→24(12:12)	TG: AA+methadone; CG: Sham AA+methadone	4 wk	1. Salivary cortisol sampled at random times among the three meals (unclear unit): 175±53 / 183±69 ($p=0.006$); 2. BSSI: 10.38±0.85 / 11.94±1.23 ($p=0.01$)	NR
Pirnia et al. (2020) / Iran / single-case experimental study	1	AA+ROC	4 wk	1. BDI: 28.51±0.64 / 26.32±0.83 ($p<0.05$); 2. BSSI: 31.12±0.37 / 25.62±0.35 ($p<0.05$); 3. Salivary cortisol	NR

				in morning (unclear unit): 17.95±0.29 / 14.74±0.24 (p<0.05)	
Li et al. (2021) / China / RCT	90(45:45)→90(45:45)	TG: EA+MT+ROC; CG: EA+ROC	1 mon	1. NGASR: 4.31±0.77 / 5.39±1.13 (p<0.05) (in TG); 7.37±1.76 to 5.39±1.13 (p<0.05) (in CG)	NR
